# Second course of stereotactic radiosurgery for locally recurrent brain metastases: Safety and efficacy

**DOI:** 10.1371/journal.pone.0195608

**Published:** 2018-04-05

**Authors:** Juliette Moreau, Toufic Khalil, Guillaume Dupic, Emmanuel Chautard, Jean-Jacques Lemaire, Florian Magnier, Véronique Dedieu, Michel Lapeyre, Pierre Verrelle, Julian Biau

**Affiliations:** 1 Radiotherapy Department, Université Clermont Auvergne, Centre Jean Perrin, Clermont-Ferrand, France; 2 Neurosurgery Department, Clermont-Ferrand Hospital, Clermont-Ferrand, France; 3 Université Clermont Auvergne, INSERM, U1240 IMoST, Clermont-Ferrand, France; 4 Université Clermont Auvergne, CNRS, UMR 6602, Institut Pascal, Clermont-Ferrand, France; 5 Medical Physics Department, Centre Jean Perrin, Clermont-Ferrand, France; 6 Radiation Oncology Department, Institut Curie, Paris, France; North Shore Long Island Jewish Health System, UNITED STATES

## Abstract

In the present study, we have evaluated the efficacy and toxicity of repeated brain metastases (BM) stereotactic radiosurgery (SRS2) following local failure of a prior radiosurgical procedure (SRS1). Between December 1996 and August 2015, 30 patients with 36 BM underwent SRS2 with a median dose of 18Gy. All BM were located outside critical structures. Following SRS2, local control at 6 months and one year were respectively 82.9% (IC 95%: 67.6–91.9) and 67.8% (IC 95%: 51–81). On multivariate analysis, planning target volume (PTV) < 3cc (HR: 0.19 (0.1–0.52)) and whole brain radiotherapy (WBRT) prior to SRS2 (HR: 0.25 (0.1–0.64)) were significantly associated with a better local control. One- and two-year overall survival rates after SRS2 were respectively 65.5% (IC 95%: 47.3–80%) and 27.6% (IC 95%: 14.7–45.7). Median overall survival following SRS2 was 14.2 months (range 1–106). Nineteen (63%) patients died from progressive systemic disease. Three (10%) patients died from out-field progressive brain disease and 8 (27%) in-field. Concerning toxicities, edema, radionecrosis, and hemorrhages were identified in 5 (12.8%), 4 (10.2%), and 5 (12.8%) patients respectively. No toxicity resulted in a neurological deficit. On univariate analysis, toxicities were significantly associated with PTV > 7cc (p = 0.02) and all patients had a WBRT before SRS2. A second course of SRS for locally recurrent brain metastases showed encouraging rates of local control. This treatment led to acceptable toxicities, especially for brain metastases smaller than 7cc, in our selected cohort of patients with BM located outside critical structures. Further studies are needed.

## Introduction

Approximately 20–40% of patients with cancer will be diagnosed with brain metastases (BM) [1]. The treatment of BM is a growing challenge due to the improvement of systemic therapies, resulting in patients with longer overall survival [2]. Whole brain radiotherapy (WBRT) has long been the standard radiotherapy modality for the treatment of BM; however, it has been associated with neurocognitive decline and decreased quality of life [3,4]. Over the last few years, stereotactic radiosurgery (SRS) has increasingly become one of the standards of care for treatment of BM [2]. SRS offers high local control rates while limiting exposure to surrounding normal tissue [5].

When local failure occurs after SRS, there is no standardized treatment option [6]. Treatment options include surgery [7,8], WBRT (if it has not already been performed) [3,9], systemic therapies [10,11], new SRS [12–15] or palliative care. A few studies have reported a variable survival time of 7–10 months for patients with recurrent BM outside the previously irradiated site who were treated with salvage SRS [14–17]. However, most of the series include patients with BM that recur after WBRT or are distant from the initial SRS site. The specific question of the safety and efficacy of a second SRS course to treat recurrence on the previously irradiated (with SRS) metastatic site remains unsubstantiated. A few studies have addressed this specific question and reported results of a median survival time of 10–26 months [12,13,18,19].

Our purpose was to evaluate the safety and efficacy of SRS as a local salvage treatment after prior failed SRS on a selected population of patients with controlled extracranial disease, no neurological deficit and BM located outside critical structures.

## Material and methods

### Population and SRS characteristics

Between December 1996 and August 2015, 341 patients were treated with SRS for 523 BM in our institution. We identified 30 patients with 36 BM initially treated with SRS (SRS1) that developed local recurrence and underwent a repeated course of SRS (SRS2) on the same site. None of the patients had a neurological deficit at time of SRS2. The selection of the patients for SRS2 followed our institutional protocol based on: i) local recurrence after SRS1 with a minimum delay of 10 months; ii) KPS ≥ 70; iii) no progression of extracranial disease; iv) maximum diameter of local recurrence of 3 cm; and v) local recurrence located outside critical structures: > 5 mm from brainstem, > 5 mm from optic nerves or chiasm and outside motor area.

From 1996 to 2010, SRS was performed with a linear accelerator Varian^®^ Clinac 2100C (Varian Medical Systems, Palo Alto, CA) equipped from 1996 to 2000 with cylindrical collimators (diameters: 6 to 24 mm) and from 2000 to 2010 with an additional micro multi-leaf collimator m3 Brainlab^®^ (Brainlab, Feldkirchen, Germany). From 1996 to 2010, a Leksell stereotactic head frame was installed under local anesthesia by a neurosurgeon. From 2010 to 2015, SRS was performed with a linear accelerator Novalis Tx^®^ (Varian Medical Systems, Palo Alto, CA) with an integrated ExacTrac X-ray 6D system^®^ (BrainLAB AG, Feldkirchen, Germany) that has the ability for pretreatment positioning. A frameless mask was made without invasive procedures.

The gross tumor volume (GTV) was identified on the basis of 0.9-mm gadolinium-enhanced axial magnetic resonance imaging (MRI) fused with high-resolution (1.25-mm slice thickness) computed tomography (CT) images. All 36 BM were treated with single fraction SRS. No margin was added to the GTV to create the planning target volume (PTV). The median prescription dose was 18Gy (range: 12-20Gy) at the isocenter with the 90% isodose covering the PTV. All treatment plans were reviewed and approved by the treating radiation oncologist, neurosurgeon and physicist.

From 2002, dose distributions were achieved using 6 MV photons with 4–5 non coplanar dynamic arcs. A conformal arc technique was used before 2002.

This study was approved by an Ethics Committee (CECIC Rhône-Alpes-Auvergne).

### Follow-up

Follow-up included MRI (T1 Gado sequences + dynamic susceptibility-weighted contrast-enhanced [DSC] perfusion) and clinical examination repeated at 3-month intervals. Last follow up was defined as the date of death for dead patients, and date of last visit with an IRM for alive patients during the period of the study that ended in August 2015. Local failures were defined as recurrences within a previously irradiated volume, according to RANO-BM criteria (relative increase of 20% in the longest diameter with an absolute value of 5 mm or more) [20] with a cerebral blood volume ratio (rCBV) ≥ 2 at DSC perfusion images [21]. Distant regional brain failures were defined as new brain metastases that were detected during follow-up imaging. The cause of death was determined in all deceased patients. Treatment-related toxicities such as radionecrosis, edema and hemorrhages were scored using RTOG central nervous system (CNS) toxicity criteria [22]. Radionecrosis was defined as stable or shrinking contrast-enhanced lesions over a 6-month observational period associated with a rCBV < 2.0 at DSC perfusion images [13,23] and/or on the basis of histologic findings (in patients who underwent surgical resection).

### Statistical analysis

Statistical analyses were performed using R v2.15.1 (http://www.cran.r-project.org). Local control rates and survival were estimated using the Kaplan–Meier method calculated from the time of SRS2. Time to progression (TTP) was defined as the period of time from SRS to the date of radiographic evidence of local progression at the treated site. Time to distant regional failure was defined as the period of time from the last date of SRS2 to the date of radiographic evidence of new brain metastases.

The optimal cut-off values have been determined using ROC curve analysis and according to clinical relevance. Univariate analysis, using the log-rank test for categorical variables and the Cox proportional hazards model for continuous variables, was performed to identify prognostic factors associated with local control, distant brain relapse, survival and toxicities. A *p* value < 0.05 was considered indicative of a statistically significant difference. The following factors were tested: tumor volume, SRS dose, interval between SRS1 and SRS2 (> or ≤1 year), sex, age (<65 vs ≥65 years), surgery before SRS, WBRT prior to SRS, pretreatment KPS score (70 vs >70), number of brain metastases (1 vs >1), diagnosis-specific graded prognostic assessment (DS-GPA) class [24], histology, and extracranial disease status. Prognostic factors with a *p* value ≤ 0.1 were included in a multivariate analysis using a Cox proportional hazards regression model.

## Results

### Patient and treatment characteristics

All characteristics of the 30 patients at the time of SRS2 are reported in Table 1. The median age was 59.3 years (range: 39–82.7), median KPS 80 (range: 70–100), and median follow-up from SRS2 13.8 months (range: 1–107). We identified 15 (50%) patients with lung cancer, 5 (17%) patients with breast cancer, 4 (13%) patients with renal cancer, and 6 (20%) patients with other primary tumors. No melanoma was reported. Six (20%) patients were DS-GPA class 0–1, five (17%), ten (33%), and nine (30%) were respectively DS-GPA score 1.5–2, 2.5–3 and 3.5–4. Of the 30 patients, 24 (80%) received WBRT and 6 (20%) never received WBRT. Of the 24 patients who received WBRT, 22 (73%) received WBRT prior to SRS1, no patient received WBRT between SRS1 and SRS2 and 2 (7%) were treated with WBRT after SRS2. WBRT dose was 30 Gy in 10 sessions for all patients. SRS2 was performed post-operatively for 3 (10%) patients. Mean PTV volume was 4.8 cc (range 0.1–24.8) at time of SRS2 *vs* 1.48 (range 0.1–18.3) at time of SRS1 (p < 0.01).

**Table 1:** Patient characteristics and treatment parameters.

Parameter	
Number of patients	30
Sex (F/M)	10 (33%) /20 (67%)
Age (years)	
Median	59.3
Range	[39–82.7]
Histology	
Lung	15 (50%)
Others*	15 (50%)
KPS	
<70	0
70	4 (13%)
80	10 (33%)
90–100	16 (54%)
Extracranial disease	
Present	10 (33%)
Absent	20 (67%)
Status of extracranial disease	
Complete response	15 (50%)
Partial response	11 (37%)
Stability	4 (13%)
Progression	0
Previous brain metastasis therapy	
Resection	3 (10%)
WBRT	22 (73%)
Number of brain metastases	
Single	20 (67%)
Multiple	10 (33%)
DS-GPA score	
0–1	6 (20%)
1.5–2	5 (17%)
2.5–3	10 (33%)
3.5–4	9 (30%)
PTVs (cc) volumes	
Range	[0.13–24.8]
Mean	6.5
Median	4.8
Dose at the PTV isocenter (Single fraction, Gy)	
Range	[12–20]
Median	18

KPS Karnofsky Performance Status, DS-GPA Diagnosis-Specific Graded Prognostic Assessment; GTV, Gross Target Volume; PTV, Planning Target Volume.

*Others histologies include breast cancer, kidney cancer, pancreas cancer, testicular cancer, ovarian cancer, rectal cancer.

### Local control

**Fig 1:**
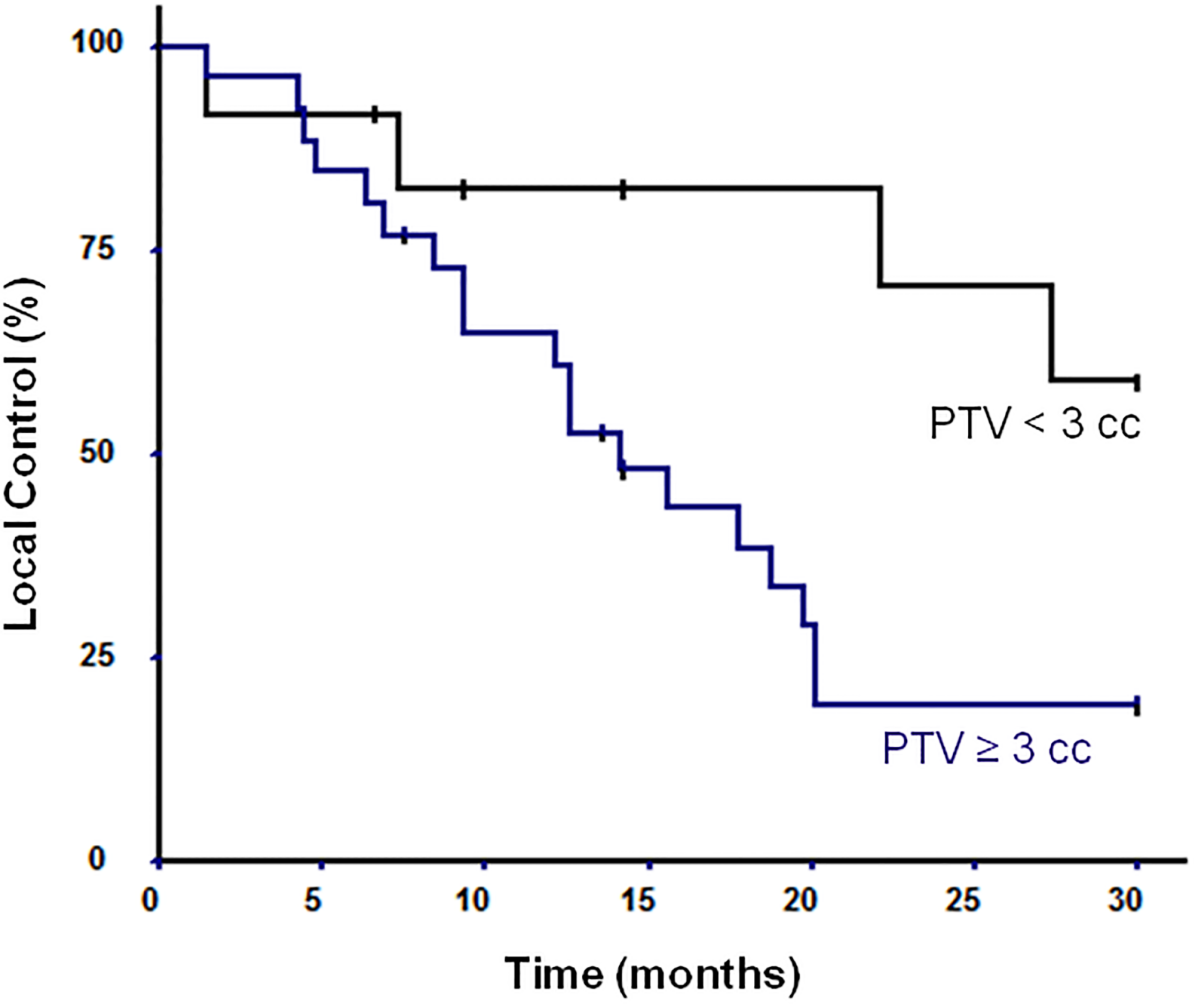
Probability of local control according to PTV volume.

**Fig 2:**
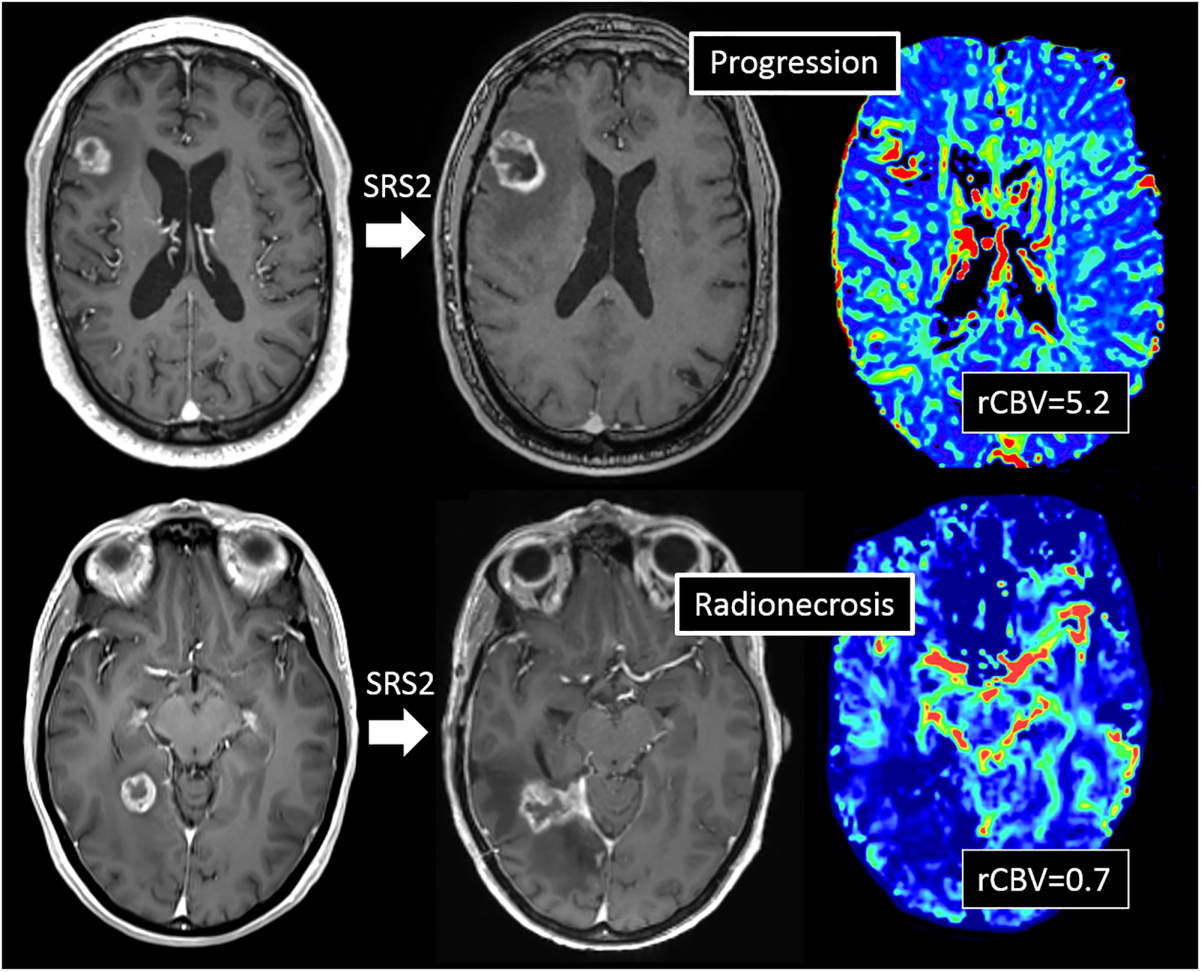
Example of brain metastasis treated with a second of radiosurgery (SRS2). Both metastasis showed an increase > 20% in the longest diameter at 9 months, one with a high cerebral blood volume ratio (rCBV = 5.2) in favor of local progression (A) and the other with low rCBV (= 0.7) in favor of radionecrosis (B).

Following SRS2, local control at 6 months and one year were respectively 82.9% (IC 95%: 67.6–91.9) and 67.8% (IC 95%: 51–81) (Figs 1 and 2). Mean TTP from SRS1 to SRS2 was 15.4 months (range 11–78). Mean TTP from SRS2 to the last follow-up was 19.7 months (range 1–106). Four (13%) patients underwent a surgical resection after SRS2 because doubt remained as to whether they had developed progression or radionecrosis. Progression was found on all patients including 2 (7%) patients presenting both radionecrosis and progression.

Results of univariate and multivariate analyses are shown in Table 2. On univariate analysis, local control after SRS2 was significantly associated with PTV < 3cc, previous WBRT and minimum dose (Dmin) to PTV > 16Gy. On multivariate analysis, PTV < 3cc (HR: 0.19 (0.1–0.52)) and WBRT prior to SRS2 (HR: 0.25 (0.1–0.64)) were significantly associated with a better local control.

**Table 2:** Factors affecting clinical outcomes after repeated stereotactic radiosurgery, on 30 patients and 36 brain metastasis.

	Univariate analysis	Multivariate analysis	
	*p* value	*p* value	HR (95%)
Local control			
Histology (*lung vs others*, *n = 15*:15)*	0.17	-	
WBRT prior to SRS2 *(Yes vs No*, *n = 22*:8)*	0.004	0.003	0.25 [0.1–0.64]
Surgery prior SRS2 *(Yes vs No*, *n = 3*:27)*	0.9	-	
Dose min *(≤ 16Gy vs > 16Gy*, *n = 14*:*22)*Ɨ	0.01	-	
Vol PTV *(< 3cc vs ≥ 3cc*, *n = 13*:*23)* Ɨ	0.003	0.003	0.19 [0.1–0.52]
Interval between SRS1/SRS2 *(< 1 year vs ≥ 1 year*, *n = 9*:21)*	0.16	-	
Regional control			
Number of brain metastases *(1 vs > 1*, *n = 20*:10)*	0.1	0.04	2.97 [1.04–8.51]
Extracranial disease *(Yes vs No*, *n = 10*:20)*	0.5	-	
WBRT prior to SRS2 *(Yes vs No*, *n = 22*:8)*	0.01	-	
Overall survival			
Histology (*lung vs others*, *n = 15*:15)*	0.6	-	
Status of disease *(complete response vs other*, *n = 15*:15)*	0.1	-	
DS GPA *(≤ 1*.*5 vs ≥ 2–2*.*5*, *n = 6*:24)*	0.8	-	
KPS *(≤ 70 vs > 70*, *n = 4*:26)*	0.8	-	
Interval between SRS1/SRS2 *(< 1 year vs ≥ 1 year*, *n = 9*:21)*	0.6	-	
Toxicities			
Dose min *(≤ 15Gy vs > 15Gy*, *n = 10*:*26)* Ɨ	0.2	-	
Long PTV *(≤ 30 mm vs > 30 mm*, *n = 10*:*26)* Ɨ	0.33	-	
Vol PTV *(< 7cc vs ≥ 7cc*, *n = 22*:*14)* Ɨ	0.05	-	

* Patients characteristics

^*Ɨ*^ brain metastasis characteristics

### Distant brain relapse

From time of SRS2, 8 (27%) patients developed distant brain metastasis. Six (20%) patients had had WBRT prior to SRS2. The mean time to distant regional failure from SRS2 was 6 months (range 1–32). Statistical analysis is shown in Table 2. On univariate analysis, distant brain relapse was significantly associated with the absence of WBRT prior to SRS2 and number of brain metastases > 1. On multivariate analysis, distant brain relapse was correlated with the number of brain metastases > 1 (p = 0.04; HR: 2.97 (1.04–8.51)).

### Survival rates and causes of death

**Fig 3:**
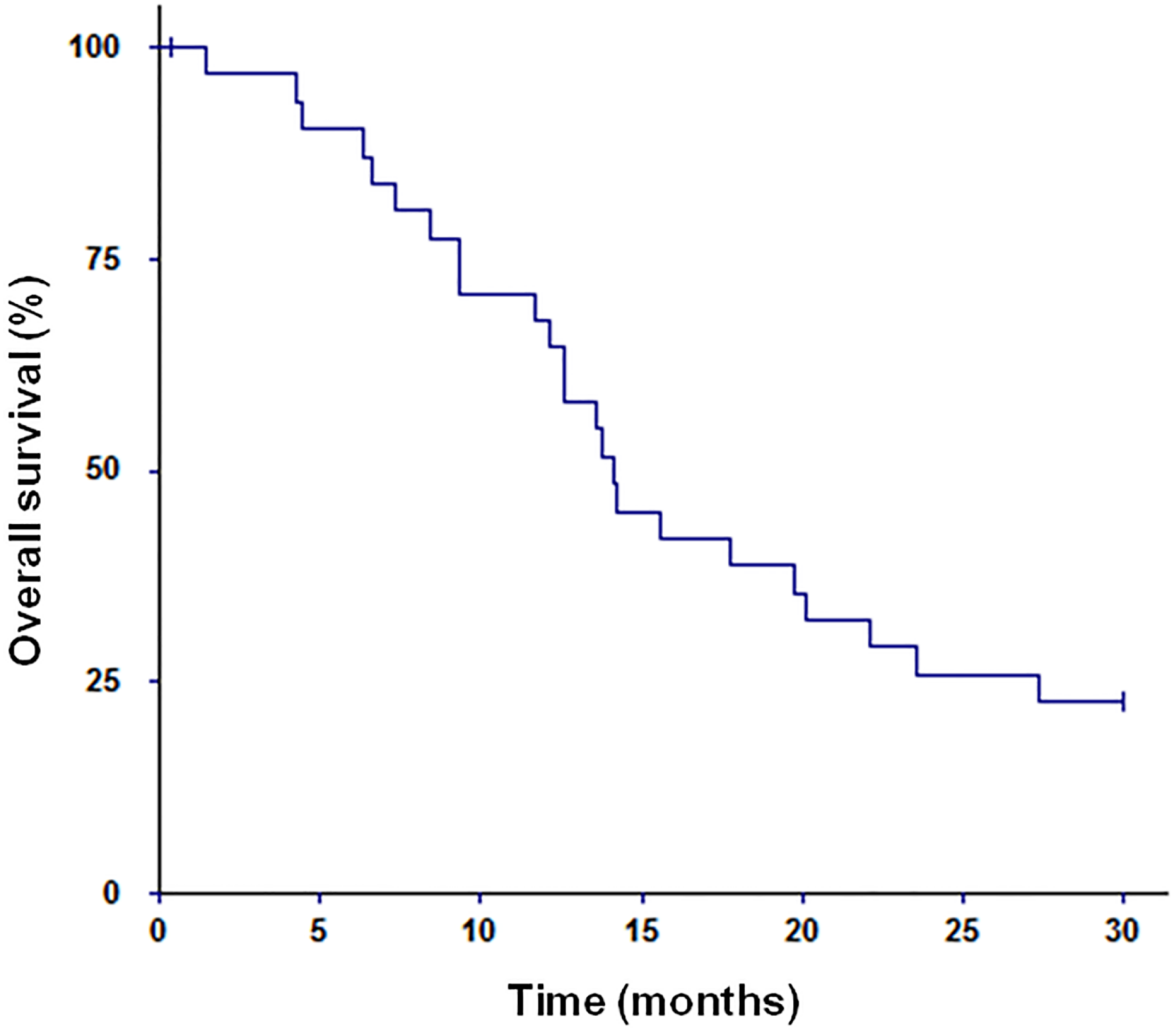
Probability of overall survival.

One- and two-year overall survival rates after SRS2 were respectively 65.5% (IC 95%: 47.3–80%) and 27.6% (IC 95%: 14.7–45.7). Median overall survival from SRS1 was 33.6 months (range 10–120). Median overall survival following SRS2 was 14.2 months (range 1–106) (Fig 3).

All patients had a clinical follow-up each three-month period with an MRI. Twenty-seven (90%) patients were dead at the last follow-up. Eleven (37%) patients presented an intracranial progression that caused death, whereas 19 (63%) had a stable neurologic disease and died from extra-neurological causes. In the group of patients who died from neurological causes (11 out of 30 patients), 8 (73%) patients died from an infield progression and 3 (27%) patients died from distant BM evolution. No deaths due to adverse effects were reported.

### Adverse effects

From time of SRS2, we identified 14 (36%) patients who presented adverse effects: radiographic local edema, radionecrosis, and hemorrhages were identified in 5 (13%), 4 (10%), and 5 (13%) patients respectively. All patients had had a WBRT before SRS2. On univariate analysis, toxicities were significantly associated with PTV volume > 7cc (p = 0.02). No neurologic deficit was observed in any of the patients who presented adverse effects. All adverse effects were scored 1 and 2 according to the RTOG toxicity criteria. No adverse effect > 2 was reported.

## Discussion

The role of retreatment in the management of recurrent/progressive BM remains challenging. Recent evidence-based clinical practice guidelines stated that since there is insufficient evidence to make definitive treatment recommendations, treatment should be individualized based on a patient’s functional status, extent of disease, volume/number of metastases, recurrence or progression at the original versus non-original site, previous treatment and type of primary cancer. Additionally, enrollment in clinical trials is encouraged [6]. Our study reports on a series of 30 patients who underwent a second course of SRS for recurrent or progressive BM. Although our study has a limited number of patients, data regarding the specific issue of repeated SRS on a BM after initial SRS failure are limited on retrospective studies, with similar sample size, between 19 and 43 patients [12,13,18,19].

We found that local control at 6 months and 1 year were respectively 82.9% and 67.8%. In the series described by Kwon et al., in which a second course of SRS was given for recurrence at previously treated sites of BM in 16 patients, local control rate at 6 months was 91% [25]. In another study by Miyakawa et al., 50 locally recurrent metastatic brain tumors in 47 patients were treated with salvage SRS after first GKS (Gamma Knife Surgery) [26]. The cumulative incidence of local recurrence after SRS was 15% at 6 months and 37% at 1 year, and the median time to local recurrence was 16 months. Our results fall within these ranges. Minniti et al. analyzed a total of 43 patients who received a second course of SRS for recurrent or progressive BM, and found 1- and 2-year local control rates were 70 and 60%, respectively [13]. They found that melanoma histology (p = 0.02), a radiation schedule of 3 × 7 Gy (p = 0.03), and large volumes (p = 0.04) were associated with worse control. In our study, through multivariate analysis, we also found that PTV volume (< 3cc) was a predictive factor of local control (p = 0.01; HR: 0.19 (0.1–0.52)). We did not find any correlation with histology; however, none of our patients had melanoma. We also found, through multivariate analysis, that previous WBRT was a predictive factor of local control after SRS2 (p = 0.003; HR: 0.25 (0.1–0.64)). This correlation was quite surprising. One can’t exclude that the durable changes induced by both WBRT and SRS1 regarding the blood-brain barrier, brain vasculature and/or cerebral endothelial cells might also have an impact of the efficacy of SRS2 [27]. Another hypothesis is that WBRT may also facilitate the crossing of systemic therapy through the blood-brain barrier permeability and may lead to a biased interpretation of better local control [28]. Other clinical, biological or statistical biases cannot be excluded.

In the studies by Kwon et al. and Minniti et al., no patients received WBRT prior to SRS2 [13,25]. Although similar survival benefits have been shown in two randomized trials between patients treated for 1–4 BM with SRS plus whole brain radiation therapy versus SRS alone [3,9], 24 patients underwent WBRT prior to SRS in our study. It could be explained by the fact that our data were mostly collected between 2000 and 2010 and that management of BM has evolved since. Using SRS alone as a first treatment for BM could potentially avoid the neurocognitive decline associated with WBRT [27]. In our study, we also found that minimum dose > 16Gy was a predictive factor of local control (p = 0.01).

We found that median overall survival was 14.2 months, which is similar to the results obtained after a first SRS. No major adverse effect was found, and no deaths resulted from treatment. Minniti et al. found a median overall survival of 10 months [13]. At multivariate analysis, stable extracranial disease and KPS > 70 were associated with a significant survival benefit. Holt et al. analyzed 13 patients who had metastatic brain lesions initially treated with definitive SRS, followed by surgical resection and a second course of SRS for recurrent brain disease [12]. They found a median overall survival of 13.3 months. Melanoma patients had significantly lower survival rates. In our study, survival was associated with the status of the extracranial disease (complete response *vs* other). We did not find any relationship between survival and KPS or DS-GPA score, which could be explained by the fact that our population was a selected population. We only had 4 (13%) patients with KPS = 70 and no patients with KPS < 70. 24 (80%) patients had a DS-GPA score ≥ 2. In the study by Miyakawa et al, the median survival time was 12 months, and the 1-year overall survival rate was 50%, which is similar to our results [26].

Radionecrosis was observed in 4 (13%) treatments which is quite similar to the results observed in the literature [28,29]. However, very few series evaluated radiation-induced toxicity after a second course of SRS on a local recurrence. In a recent study, McKay et al. studied forty-six lesions in 32 patients treated with a second course of SRS after local failure and found that freedom from radiation necrosis at 1 year was 71% (95% CI 57%-88%) and that the V40Gy was predictive of radiation necrosis [30]. Minniti et al. found that radionecrosis occurred in 19% of treated lesions after a second course of SRS given in three fractions and was associated with severe neurological complications (RTOG Grade 3 or 4) in 14% of patients [13]. Holt et al [12] found 13.3% of grade ≥ 2 radionecrosis with a clinical impact on patients who underwent repeated SRS after tumor resection. Miyakawa et al. found only two patients who had developed ≥ grade 2 radiation necrosis on 47 patients [26]. For local edema, all imaging was analyzed and compared so we could define whether they were due to SRS or to the lesion. In a related study, Hanna et al. found a local edema in 18% of patients after SRS on 114 metastases [31]. Radiographic local edema was found in 13% of cases in our study, which was similar to these results. The author suggested that melanoma/renal histology, recursive partitioning analysis class III, and prior WBRT carried relative risks of developing post-SRS edema: an increase of 2.45, 2.48, and 3.16, respectively. No such results have been shown in our study, probably because of the low numbers of patients. In the study by Trifiletti et al., 24% of patients developed local edema after SRS2 [19]. Miyakawa et al. found symptomatic radiation-induced edema in 51% of patients, but in most cases corticosteroids were clinically efficient [26]. Risks of hemorrhage have been mostly studied for melanoma and renal cancer and could vary from 10% to 25% [32,33]. In our study, there were four renal cancers and no melanoma. However, we found a 13% occurrence of hemorrhage, which falls within this range. Overall, we have to highlight that no serious neurological side effects were found in our study, certainly because all our patients were selected with BM outside critical structures: > 10 mm from brainstem, > 10 mm from optic nerves or chiasm and outside motor area. As we found that toxicities were mostly associated with BM volume (PTV ≥ 7cc vs < 7cc; p = 0.05), care should be taken in that particular situation. Hypofractionated stereotactic radiotherapy (usually 3–5 fractions) might be a better option for the largest BM, as hypofractionation has a potential benefit against radiation-induced toxicity [13,34]. However, further studies are needed.

## Conclusion

A second course of SRS for locally recurrent brain metastases showed encouraging rates of local control, especially for brain metastases < 3cc. The risk of toxicity was moderate, especially for brain metastases < 7cc. No neurological side effects were found in our selected cohort of patients with brain metastases outside critical structures (> 10 mm from brainstem and optic structures and outside the motor area). Further investigations are needed to optimize the management of recurrent brain metastases after initial SRS, and prospective studies with a larger population should be carry out.
